# Implementing surgical mentorship in a resource-constrained context: a mixed methods assessment of the experiences of mentees, mentors, and leaders, and lessons learned

**DOI:** 10.1186/s12909-022-03691-2

**Published:** 2022-08-31

**Authors:** Shehnaz Alidina, Meaghan M. Sydlowski, Olivia Ahearn, Bizuayehu G. Andualem, David Barash, Sehrish Bari, Erin Barringer, Abebe Bekele, Andualem D. Beyene, Daniel G. Burssa, Miliard Derbew, Laura Drown, Dereje Gulilat, Teruwork K. Gultie, Tuna C. Hayirli, John G. Meara, Steven J. Staffa, Samson E. Workineh, Noor Zanial, Zebenay B. Zeleke, Abraham E. Mengistu, Tigistu A. Ashengo

**Affiliations:** 1 Program in Global Surgery and Social Change, Harvard Medical School, 641 Huntington Avenue, MB Boston, USA; 2 Amhara Regional Health Bureau, Federal Ministry of Health, Addis Ababa, Ethiopia; 3 GE Foundation, Boston, MA USA; 4 D-Implement, Dalberg Advisors, New York, USA; 5 College of Health Sciences, Addis Ababa University, Addis Ababa, Ethiopia; 6 Federal Ministry of Health, Addis Ababa, Ethiopia; 7 Safe Surgery 2020, Project, Jhpiego, Addis Ababa, Ethiopia; 8 Department of Plastic and Oral Surgery, Boston Children’s Hospital, Boston, MA USA; 9 Department of Pediatrics, University of Melbourne, Melbourne, Australia; 10 Department of Anesthesiology and Surgery, Boston Children’s Hospital, Boston, MA USA; 11 Safe Surgery 2020, Jhpiego, Baltimore, MD USA

**Keywords:** Mentorship, Surgery, Ethiopia, Implementation, Safe Surgery 2020, Workforce

## Abstract

**Background:**

A well-qualified workforce is critical to effective functioning of health systems and populations; however, skill gaps present a challenge in low-resource settings. While an emerging body of evidence suggests that mentorship can improve quality, access, and systems in African health settings by building the capacity of health providers, less is known about its implementation in surgery. We studied a novel surgical mentorship intervention as part of a safe surgery intervention (Safe Surgery 2020) in five rural Ethiopian facilities to understand factors affecting implementation of surgical mentorship in resource–constrained settings.

**Methods:**

We designed a convergent mixed-methods study to understand the experiences of mentees, mentors, hospital leaders, and external stakeholders with the mentorship intervention. Quantitative data was collected through a survey (*n* = 25) and qualitative data through in-depth interviews (*n* = 26) in 2018 to gather information on (1) intervention characteristics including areas of mentorship, mentee-mentor relationships, and mentor characteristics, (2) organizational context including facilitators and barriers to implementation, (3) perceived impact, and (4) respondent characteristics. We analyzed the quantitative and qualitative data using frequency analysis and the constant comparison method, respectively; we integrated findings to identify themes.

**Results:**

All mentees (100%) experienced the intervention as positive. Participants perceived impact as: safer and more frequent surgical procedures, collegial bonds between mentees and mentors, empowerment among mentees, and a culture of continuous learning. Over 70% of all mentees reported their confidence and job satisfaction increased. Supportive intervention characteristics included a systems focus, psychologically safe mentee-mentor relationships, and mentor characteristics including generosity with time and knowledge, understanding of local context, and interpersonal skills. Supportive organizational context included a receptive implementation climate. Intervention challenges included insufficient clinical training, inadequate mentor support, and inadequate dose. Organizational context challenges included resource constraints and a lack of common understanding of the intervention.

**Conclusion:**

We offer lessons for intervention designers, policy makers, and practitioners about optimizing surgical mentorship interventions in resource-constrained settings. We attribute the intervention’s success to its holistic approach, a receptive climate, and effective mentee-mentor relationships. These qualities, along with policy support and adapting the intervention through user feedback are important for successful implementation.

**Supplementary Information:**

The online version contains supplementary material available at 10.1186/s12909-022-03691-2.

## Background

Designing and implementing surgical mentorship is a novel component of efforts to strengthen surgical systems in low- and middle-income countries (LMICs). Lack of training is partially responsible for the scarcity of surgical, obstetric, and anesthesia providers in LMICs [1, 2]. In 2015, the Lancet Commission on Global Surgery placed surgical workforce development on top of the health, welfare, and economic development agenda [3]. Building the capacity of surgical systems through mentorship is a promising strategy to improve access and quality of care [4, 5]. However, implementation of surgical mentorship in African healthcare settings has not been thoroughly analyzed [6]. In this paper, we examine factors affecting implementation of the Safe Surgery 2020 (SS2020) mentorship intervention in Ethiopia to draw insights about optimizing surgical mentorship in resource-constrained settings.

Ethiopia is a country of approximately 109 million people and ranks 173 out of 189 countries on the Human Development Index [7]. Its low surgical provider density (0.6 specialist surgical providers per 100,000) contributes to one of the lowest surgical rates in the world – 148 surgeries per 100,000—with most specialists based in large cities [8]. Much of the burden of emergency and essential surgery is placed on lower-level hospitals. In 2010, the Ethiopian Federal Ministry of Health (FMOH) introduced Integrated Emergency Surgical Officers (IESO) to handle emergency obstetric, gynecological, and surgical cases at primary hospitals and health centers [9].

Responding to unmet surgical needs, the FMOH pioneered Saving Lives through Safe Surgery (SaLTS) in 2015 [9], making Ethiopia one of the first LMICs to nationally prioritize access to safe, essential, and emergency surgical care. A baseline assessment of surgical capacity in Ethiopia revealed that, besides having a large unmet burden of surgically treatable disease, many health facilities faced difficulties providing essential services due to various limitations, including shortage of surgical, anesthesia, and obstetric clinicians [10] A key pillar of the SaLTS initiative, therefore, involved developing and implementing a national leadership and surgical mentorship program [11].

Mentorship is a common professional development process across industries and professions [12]. The National Academies of Sciences, Engineering, and Medicine define mentorship as a relationship “in which individuals work together over time to support the personal and professional growth, development, and success of the relational partners through the provision of career and psychosocial support” [13]. Mentorship can range from dyadic to team-based, formal to informal, peer to hierarchical, co-located to virtual, among other dimensions [14, 15]. In Africa, mentorship interventions have embedded mentors in health facilities, deployed visits by mobile mentors, involved teams of mobile multidisciplinary mentors, coupled facilities, and appointed focal mentors within facilities [6]. Mentoring can supplement—and is distinct from—coaching, advising, teaching, tutoring, advocacy, sponsorship, and role modeling, in that mentorship involves building complex, emotionally deep, and longitudinal alliances [15, 16]. In healthcare, mentorship is known to influence personal and professional development, as well as research productivity [17]. Desirable surgical mentor characteristics include “acting as a professional role model, being supportive of and involved in the trainee’s progress, serving as a trusted evaluator of the mentee, and being a leader in their field” [18]. Mentees prefer mentors with an interest in them as a mentee and those who have gone through mentoring training [19]. While coaching is a validated method to enable surgeons to acquire both technical and non-technical skills in high-income countries [20], surgical mentorship in LMICs has received less attention, with studies calling attention to the need to address inadequate mentorship [21].

Studying surgical mentorship in the LMIC context matters because of differences from high-income settings in training culture, availability of mentors, institutional resources and support, and differences in geographic, environmental, and social contexts that may constitute unique illnesses [22, 23]. Mentorship in LMICs is inequitably hindered by challenges including brain drain, lack of innovation hubs and platforms, and funding at the systems level [24]. These differences are important for adoption and institutionalization of mentorship among the surgical workforce. For example, a study of mentorship interventions in five sub-Saharan African countries found that contextually adapting mentorship and coaching interventions was necessary to improve management quality and skill transfer [25]. Similar studies include a variety of approaches to mentoring – including mobile, virtual, and within-facility mentorship – to target improvements in laboratory management, maternal and child health services, infectious disease management, cataract surgery, and managerial performance [6]. More research is needed on the role of surgical mentorship in improving access to safe and high-quality care in LMICs.

SS2020 is a global collaborative initiative that aims to strengthen surgical systems in LMICs through capacity building. The SS2020 mentorship intervention in Ethiopia was designed with government and local stakeholders to aid the development of technical and non-technical skills among surgical, anesthesia, and obstetric providers. We studied the SS2020 mentorship intervention to understand factors affecting implementation. By analyzing the experiences of mentees, mentors, hospital leaders, and key stakeholders with the SS2020 mentorship intervention, our study aims to generate learnings about how to optimize surgical mentorship in LMICs.

Our conceptual framework draws upon prior research on factors associated with effective implementation of innovations [26–30] and mentorship interventions in LMICs [31–37], and our own experience with the mentorship intervention. We focused on two dimensions of implementation—intervention characteristics and organizational context—because they are important in the surgical setting and amenable to modification. To analyze the data, we identified three sub-questions: 1) What characteristics of the intervention provided support and what factors posed challenges? 2) What are the supportive and challenging factors related to the organizational context? and 3) What is the perceived impact of the mentorship program?

## Mentorship intervention

In 2017, SS2020 introduced a multicomponent intervention to improve access to safe, high-quality surgical care in five rural hospitals in Tigray. SS2020 included three components – (1) leadership training; (2) safety protocols, data quality training, and an infrastructure grant; and (3) mentorship [7]. The aims of mentorship were to build the capacity of surgical teams, problem-solve with limited resources, and strengthen the surgical ecosystem.

SS2020 collaborated with the Surgical Society of Ethiopia, FMOH, and Regional Health Bureau (RHB) to design the intervention and conduct on-site visits, and aligned with the Ethiopian Hospitals Alliance for Quality’s hospital clusters for sustainability [8]. Initially, one mentor coordinator and one mentor per hospital were recruited from Ayder Regional Hospital based on reputation, clinical experience, and interpersonal skills. Mentors from the Surgical Society of Ethiopia and the Ethiopian Society of Anesthesiologists provided backstopping and supportive supervision. The mentor training curriculum included a week-long leadership session with mentees and two days of mentorship training. Following leadership training, mentees developed a quality improvement plan targeting surgical volume and safety in their facilities. Based on early experience, the mentorship team evolved to a multidisciplinary model [38].

The typical mentor visit was one day per month where mentors: (1) discussed priorities with hospital management; (2) reviewed progress on surgical improvement plans with mentees, discussed challenges, and answered questions; (3) provided side-by-side mentorship on clinical procedures, safety and teamwork practices, and data monitoring, and problem-solving through resource mobilization and patient consultations; (4) identified gaps and discussed next steps; (5) debriefed with hospital management; (6) and reported to the RHB.

Early visits focused on establishing relationships and trust between mentors and mentees, then developing technical and non-technical skills in mentees and advocating to hospital management and RHB for resources. Monitoring implementation included a standardized checklist to track activities and outcomes such as surgical volume, Surgical Safety Checklist (SSC) use, complications, and postsurgical infections.

## Methods

### Study design

We used a convergent mixed-methods study design [39–41] to understand the factors affecting implementation of a surgical mentorship intervention. Quantitative data were collected through surveys to understand experiences with the mentorship intervention. Qualitative data were collected using in-depth interviews to expand our understanding and gather contextual and implementation information. We received ethical approval from the Ethiopian Public Health Institute and the Institutional Review Board at Harvard Medical School reviewed the project and gave it an exemption determination. We followed reporting guidelines for qualitative research [42].

### Setting

Our setting was Tigray, Ethiopia, selected by the FMOH for SS2020 intervention. The RHB and Jhpiego selected five rural hospitals based on their commitment to quality improvement and proximity to Ayder Regional Hospital. Surgery was provided by non-specialist providers including IESOs, nurse anesthetists, and nurses.

### Participants

We used an information-rich [43], purposeful sample [44] of participants with direct mentorship intervention experience. Our sample included (1) surgical team mentees, including IESOs, anesthetists, and nurses; (2) a multidisciplinary mentorship team including a general surgeon, an obstetrician/gynecologist (as required), an orthopedic surgeon (as required), an anesthetist, a senior OR nurse, and the mentor coordinator; (3) hospital leaders including hospital administrators or medical directors; and (4) key stakeholders involved in the intervention design who could provide history and context.

The eligible sample included: 3 surgical providers and 1.2 anesthetists per hospital (on average); 1 administrator and 1 medical director per hospital; 5 mentors and 1 mentor coordinator; and 3 key stakeholders. There were no dedicated OR nurses as they rotated from the hospital wards. Our sample size was guided by information power [45]. We aimed to interview 2–3 surgical team members, 1 hospital leader, 3 mentors and a mentor coordinator, and 3 stakeholders.

### Data collection and analysis

#### Data collection

We administered a paper-based survey to mentees and hospital leaders onsite 32 months after initiation of the intervention to ensure sufficient exposure to the intervention. The survey was based on mentorship literature in African settings and innovation implementation in healthcare, developed by the research team (SA, MS, AE, SW) in consultation with stakeholders, and pilot-tested in five facilities in Ethiopia. The 57 items focused on: (1) intervention characteristics including areas of mentorship, mentee-mentor relationships, and mentor characteristics (2), organizational context including facilitators and barriers to implementation (3), perceived impact, and (4) respondent characteristics (Additional File 1). Hospital leaders and surgical team leaders identified the participants. We did not collect identifiers or offer incentives for completion.

We conducted in-depth, semi-structured, one-on-one interviews 32 months onsite after initiation of the intervention. Interview protocols for mentees, mentors, leaders, and stakeholders explored (1) intervention characteristics including which areas of mentorship were most valued and mentee-mentor relationships, (2) facilitators and barriers to implementation, and (3) perceived impact (Additional File 2). We invited all survey participants to interview, however 6 could not participate due to time constraints. In addition, we interviewed mentors and key stakeholders. Interviews were approximately 45–60 min and conducted in a private space in English by a senior researcher (SA) and an Ethiopian research team member (SW) who provided Tigrinya translation. SA has a doctoral degree in health policy and management with experience in implementation and qualitative research. Stakeholder interviews were conducted by telephone by a research assistant (MS, OA or LD) who hold master’s degrees in public health with experience in qualitative research.

Interviews were conducted individually except for 1 interview conducted with 2 interviewees due to their schedule constraints. Participants provided verbal consent before interviews. No interviewees refused or dropped out of interviews. We documented our observations and insights in field notes. Interviews were audiotaped and transcribed. Transcripts were quality checked and uploaded to NVivo V.11 (QSR International, Melbourne, Australia) for coding.

#### Data analysis

Descriptive statistics were used to summarize the percentage of responses on the Likert scale for each survey question. The denominator for each response was the number of responses to the question. Given the sample size (*n* = 25), statistical testing was not conducted. All analyses were performed using Stata (version 16.0, StataCorp LLC. College Station, TX).

We analyzed qualitative data inductively to understand factors (supportive and challenging) affecting implementation related to (1) intervention characteristics and (2) organizational context. We also examined perceived impact. First, two researchers (MS and OA) reviewed three transcripts to develop a preliminary codebook inductively based on our conceptual framework [46, 47]. The coding team had discussions to arrive at a unified codebook and used it to code nine new transcripts. Codes were validated against new text and refined until no new codes emerged (i.e. code saturation) [48]. Inter-rater reliability between two coders found substantial agreement (kappa = 0.76) [49], and all 26 transcripts were analyzed using the final codebook. Three researchers (SA, MS, and NZ) reviewed coded data, inductively extracted emerging themes based on categorization and connectively between codes, and finalized themes including minor themes [50–52], through iterative discussions.

The qualitative and quantitative data were integrated to identify patterns and themes. Coauthors reviewed themes and identified lessons emerging about factors affecting implementation of a surgical mentorship intervention in low-resource settings.

## Results

Hospital and respondent characteristics are presented in Table 1. The typical hospital was a rural hospital performing on average 33.8 surgeries per month. Survey respondents (*n* = 25) included mentees (*n* = 20, 80%) and hospital leaders (*n* = 3; 12%). Interviewees (*n* = 26) included mentees (*n* = 16/26, 61%), mentors (*n* = 4/26, 15%), hospital leaders (*n* = 3/26, 12%), and external stakeholders (*n* = 3/26, 12%).

**Table 1:** Characteristics of intervention hospitals and participants, 2018

**Hospital Characteristics (*N* = 5) n (%)**	
**Level of hospital**	
Primary Hospital	2 (40%)
General Hospital	3 (60%)
**Geography**	
Rural	5 (100%)
**Number of inpatient beds**	
0–100	3 (60%)
101–300	2 (40%)
**Average monthly surgeries per hospital**	33.8
Bellwether procedures	
Cesarean delivery	15.4
Laparotomy	4.4
Open fracture repair	0.4
Elective surgeries	13.6
**Average number of surgical providers per hospital**	
General surgeons	0.6
Obstetricians/Gynecologists	0.4
Anesthesiologists	0.0
IESOs	2.0
Anesthetists	1.2
**Participant Characteristics**	
*Survey (N* = *25) n (%)*	
**Role**	
IESO (surgical provider)	7 (28%)
Anesthetist	2 (8%)
Nurse	11(44%)
Hospital leader	3 (12%)
Other (missing)	2 (8%)
**Years in role**	
< 1 year	2 (8%)
1–3 years	5 (20%)
> 3 years	18 (72%)
**Present for mentorship visits**	
≤ 3	6 (24%)
> 3	19 (76%)
*Interviews (N* = *26) n (%)*	
**Role**	
Mentees	
IESO (surgical provider)	7 (26.9%)
Anesthetist	2 (7.7%)
Nurse	7 (26.9%)
Hospital leader	3 (11.5%)
Mentors	4 (15.4%)
Key stakeholders	3 (11.5%)

Below, we present our quantitative and qualitative results, including 3 themes and 16 constituent subthemes. Table 2 provides quotations illustrating our themes, edited for conciseness.

**Table 2:** Illustrative quotations on factors that support and challenge the mentorship intervention and perceived impact

Themes and sub-themes	Illustrative Quotations^a^
**Factors supporting implementation**	
***Intervention characteristics***	
Systems focus of the mentorship intervention	*The goals are to improve surgical outcomes and to practice safe surgery. So in that work there are different things, like improving surgical techniques, surgical site infections, safe anesthesia, and safe instrument handling, and also increasing surgical volume.* (*Hospital 4, Surgical Team Leader)*
Multidisciplinary mentorship team	*So, we went there but after a couple of visits, we modified it. We saw that there was a very big gap that we couldn't fill… We thought that it's not fair to only involve the surgeons and scrub nurses. It's good to have a team which involves a gynecologist, orthopedic surgeon, anesthesiologist, and scrub nurses. (Mentor)*​​
Psychological safety	*They are not judging. They try to make sure we don’t repeat [mistakes]. There is no blame. There is no shame. Even though we have some mistakes, they don’t want to show us. They just told us, ‘See what will be the next outcome.’ Directly they tell us the next outcome will be bad. That means we are missing something. So just we understood them clearly without any trauma for us. (Hospital 5, Surgical Team Leader)* *They are easy, safe to communicate with …even if you do wrong…This is why I like it. They teach like friends. (Hospital 1, Nurse)*
Mentor characteristics	
-Generosity	*X-ray, ultrasound, one mentor privately helped us, 17,000 birr. Out of [their] own pocket. And if we have any problem with an operation, we’ll contact with phone. (Hospital 5, IESO)*
-Accessibility	*One is the team approach because we are consulting them at night, during the day, on holiday, at any time. If we find any challenge, if we need to contact them, they are ready. Even if they are in a meeting or unable to speak with us, they text us a message. (Hospital 1, IESO)*
-Understanding of local context	*We shouldn’t say “You have to do this, you don’t have to do this”, but understand why they do it. Is it because of the lack of knowledge, skills, experience, infrastructure? … So, we were trying to understand their challenges, their gaps, and their reasons for doing things. (Mentor)*
-Interpersonal skills	*We trust them. They come, they are open, they are honest, they speak frankly. (Hospital 4, Surgical Team Leader)*
*** Organizational context***	
Receptive implementation climate	*In our hospital mentorship is so good. We [leaders] are so eager to be mentored too because we [also] get support from this, and improvement. So, there is good support by hospital management, there is good support and willingness to be mentored by the surgical team. (Hospital 4, Medical Director & Obstetrician/Gynecologist)*
**Challenges to implementation**	
***Intervention characteristics***	
Insufficient clinical training	*OR nurses [should] be trained as OR nurses. Clinical training. Some of them are lacking surgical knowledge. There is no surgery without surgical nurses. (Hospital 2, IESO)*
Inadequate mentor support	*Engagement, ownership of mentorship by Regional Health Bureaus, by people in the Ministry that needs to really improve. There is a perception that mentorship is an extracurricular activity provided by senior physicians for free, so this attitude about mentorship has really to change. (Key Stakeholder)*
***Organizational context***	
Challenging implementation context	*Training is not enough. There are also some supplies which [are not available]. There are many interruptions due to many problems. They are aiming to increase surgical volume, but they have a single anesthetist, which sometimes gets sick and can't work. (Hospital 4, Surgical Team leader)*
Lack of clear understanding of the intervention	*The mentees expect a lot of things from us. We cannot say that we met their needs. There are a lot of things, equipment, even their engagement in higher education, even in referral hospitals. Because of the financial constraints here, we cannot access these things. (Mentor)*
**Perceived impact of mentorship program**	
Safer and more frequent provision of surgical care	*The checklist was not filled before, and now they fill it after the mentorship, and surgical site infections – they did not have a uniform way of registering it. It was haphazard but now they have registers in the wards for the surgical site infections. (Hospital 4, Nurse)*
Establishment of collegial bonds between mentees and mentors	*If they have challenge, or problem in the OR theatre, they call, and they [mentors] will solve their problems. I remember one very complicated surgery, so they called Dr. [name], a known gynaecologist from [site]. So, we were calling [them and they were] guiding us by phone. They were directing us. (Hospital 1, Medical Officer)*
Empowered mentees	*Previously [the mentees] tended to externalize things, “So because we don’t have this, we don’t have this, and we don’t have this”, – something like that. So, by visiting them frequently and giving them their presentations, they realize that the majority of the things can be done within their sphere. So, I think that’s the impact we found out. They realize that they can solve by themselves, and most of the things can be done by simple interventions by themselves. (Mentor)*
Inculcation of a culture of continuous learning	*A surgical site infection was not considered as a problem before—just patients are treated for surgical site infection but there was not any reason to describe how the infection was happening. But currently, we discuss if a patient post operatively will have a surgical site infection, just what is the reason. Is it from the surgeon? From the sterility technique? From the patient themselves? Or from the OR materials? We are discussing so we are saving patients from dying by infection or by sepsis. (Hospital 5, Surgical Team Leader)*​

^a^Quotations have been edited for conciseness

### Factors supporting implementation

#### Intervention characteristics

##### Systems focus of the mentorship intervention

Interview participants noted mentorship took a holistic approach by improving technical and non-technical skills and strengthening the surgical ecosystem. For example, to improve surgical quality, mentors trained mentees on safety, teamwork, and communication practices on the SSC and coached them in the OR (operating room). To reduce inappropriate referrals out, mentors introduced a register to track surgeries and referrals and devised an action plan with strategies including mentoring on a greater range of surgeries, mobilizing resources, and communicating with mentees regarding inappropriate referrals.

**Fig. 1:**
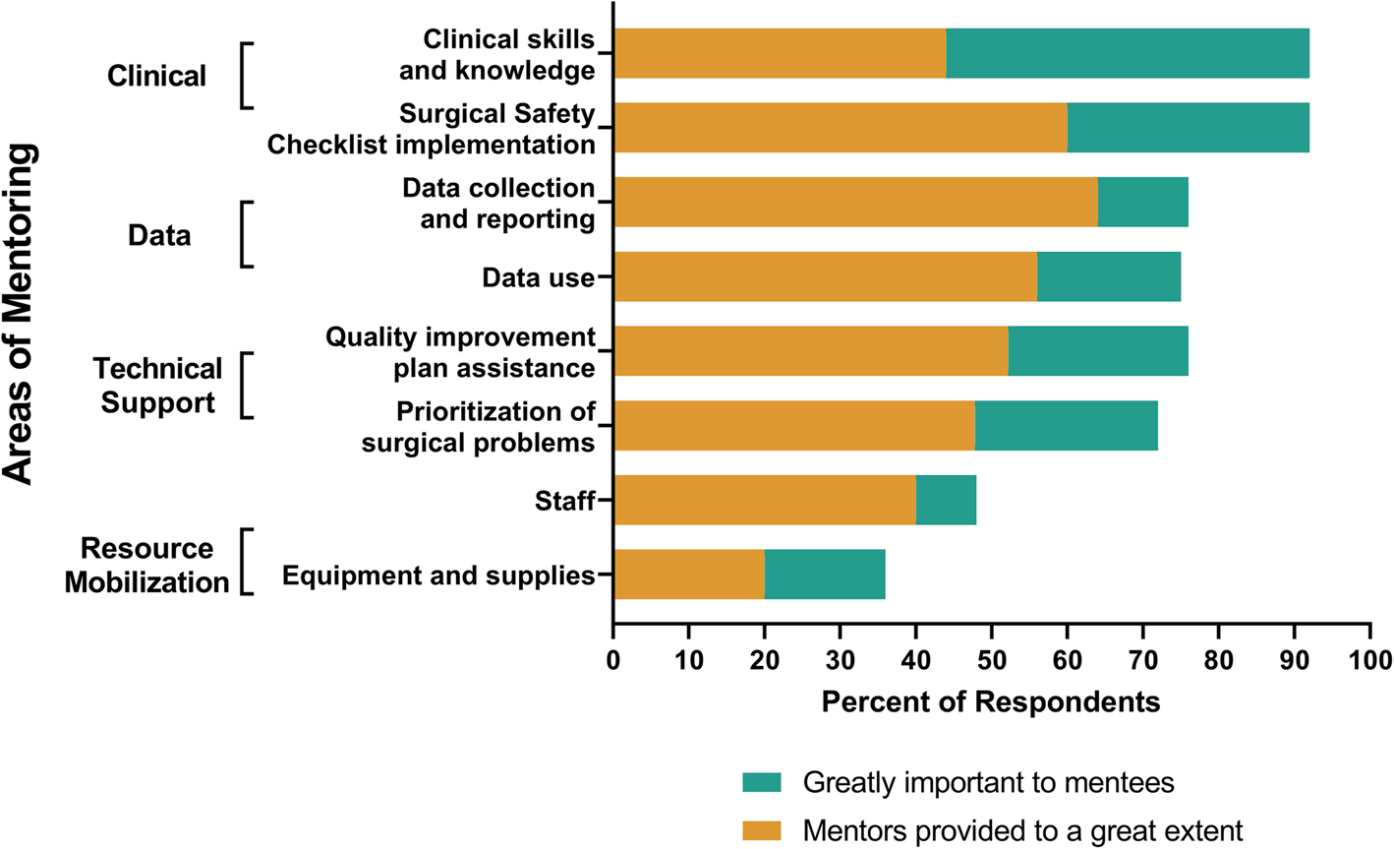
Areas of Mentorship—Prioritized and Received. Mentees prioritized clinical skills and knowledge the most. It was also the area of most discrepancy between what mentees prioritized and received—92% identified it as “greatly important” but only 44% reported it was received “to a great extent”

Mentors worked to strengthen the surgical ecosystem by providing technical expertise and advocated for equipment, staffing, water, and electricity needs to the RHB. In surveys, there was less discrepancy between what mentees desired and received on staff mobilization: 48% (*n* = 12/25) said it was greatly important and 40% (*n* = 10/25) said it was received to a great extent. There was more discrepancy regarding resource mobilization: 36% (*n* = 9/25) said it was greatly important and 20% (*n* = 5/25) said it was received to a great extent (Fig. 1).

##### Multidisciplinary mentorship team

A topic discussed less frequently but with high concern from participants was the importance of a multidisciplinary mentorship team. Interviewees stated mentorship began with one mentor per hospital, so surgical providers also mentored anesthetists and nurses. Nurse mentees reported feeling left out and unsatisfied because their profession was not well represented in the mentorship team, therefore mentorship did not address their issues adequately. Over time, the model shifted to a multidisciplinary team. Mentees expressed a high level of satisfaction with this approach.

##### Psychological safety

Mentees consistently reported relationships were collaborative and non-hierarchical; mentors approached them as friends. Mentors gave nonjudgmental feedback and had a no blame, no shame attitude. For example, mentees explained they now track the number of surgical site infections, but before mentorship it was considered shameful. Leaders noted friendly, nonjudgmental, and safe relationships created receptiveness to feedback and an environment of learning.

##### Mentor characteristics

Mentor characteristics were important to mentees. In survey results, over 80% of mentees identified ‘courtesy and respect’, ‘belief in capabilities of mentees’, ‘knowledgeable in the field’, ‘trust’, ‘constructive feedback’, ‘interpersonal skills’, and ‘role model in providing surgical services’ as “greatly important” qualities in an effective mentor (Additional File 3). Four sub-themes emerged in interviews.

##### Generosity with time, knowledge, and money

Mentees and leaders repeatedly noted mentors’ generosity with time, knowledge, and money. Mentors were highly respected senior specialists with heavy workloads but used their knowledge to teach and encourage mentees to improve access to safe, high-quality surgical care. Mentors were also generous with their financial resources, sometimes purchasing equipment with their own money.

#### Accessibility

Mentees and leaders described they valued accessibility of mentors outside their regular visits. Mentors followed up on resolving problems, advised patient consultations and referrals, and guided difficult cases. Mentees appreciated that mentors responded to their calls regardless of the time. One mentee recalled how their mentor guided them step-by-step over the phone during an operation on a complicated case.

##### Understanding of local context

A less common but notable theme was understanding of local context. Mentors and key stakeholders discussed the importance of understanding the region’s culture, hospital infrastructure, and outcomes. One mentor mentioned the need to understand the internal context of facilities and why providers practiced in certain ways.

##### Interpersonal skills

Mentees and leaders emphasized mentors’ interpersonal skills. They described them as kind, good listeners, and having integrity. Mentees explained they trusted their mentors because they were open, honest, and spoke candidly.

#### Organizational context

##### Receptive implementation climate

Mentees, mentors, and leaders commonly expressed how mentees desired to improve access to surgical services and patient outcomes. They were eager for mentors to perform procedures with them, provide feedback, discuss cases, and problem-solve. Hospital leaders valued mentors’ role in promoting learning, mobilizing resources, and advising. One mentee explained that from their perspective, 90% of the mentees were motivated, but 10% were not because mentorship created additional work without incentives.

### Challenges to implementation

#### Intervention characteristics

##### Insufficient clinical training

**Fig. 2:**
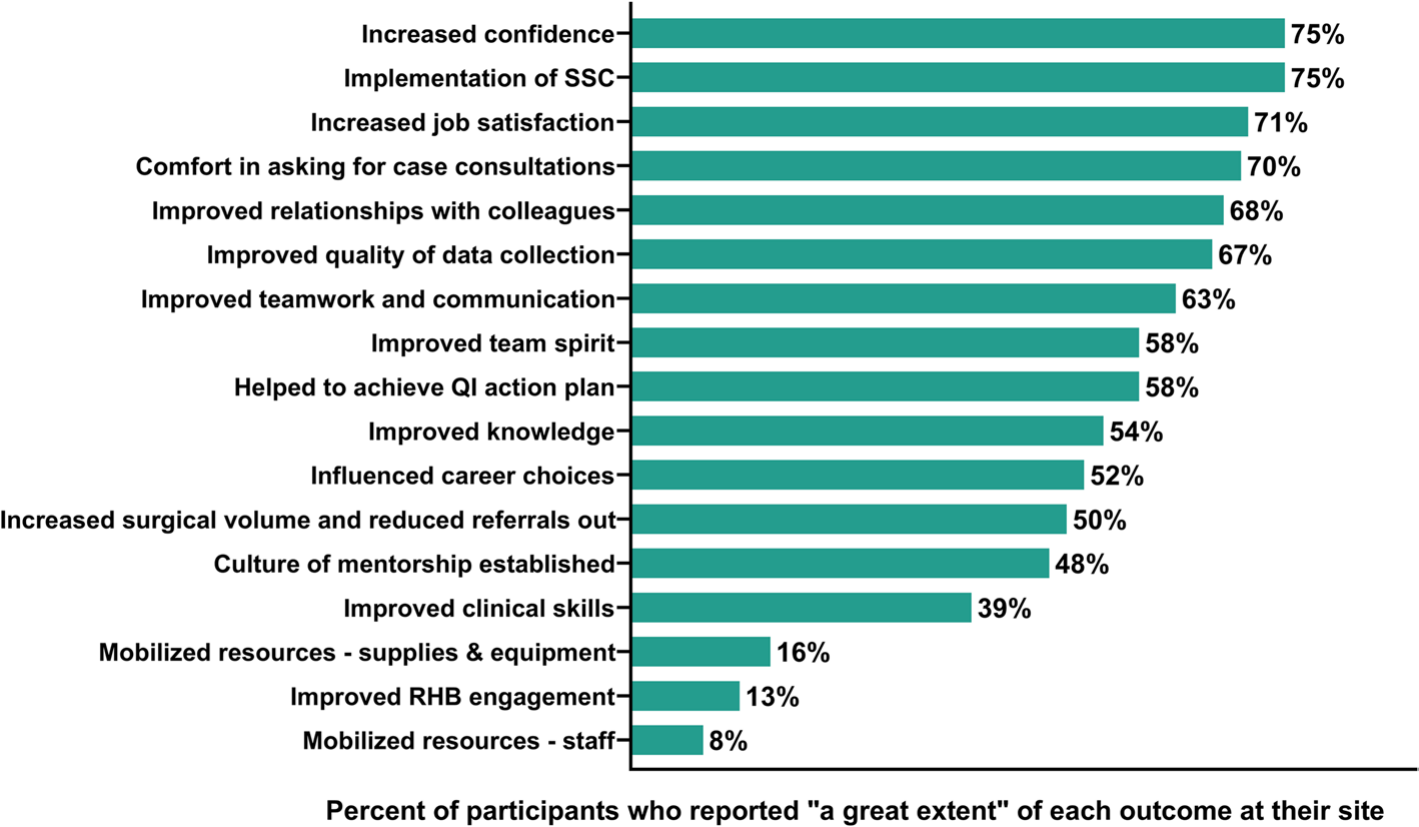
Perceived Impact of the Mentorship Intervention. Over 70% of mentees reported three areas that had improved ‘to a great extent’: increased confidence (75%), SSC implementation (75%), and improved job satisfaction (71%)

In surveys, the largest reporting discrepancy was in clinical mentorship: 92% (*n* = 23/25) identified it as “greatly important” but only 39% said their clinical skills improved “to a great extent” (Fig. 2). Mentees discussed the duration of mentorship visits, emergencies, and fluxes in surgical volume hindered time for side-by-side surgical mentorship. Some mentors and key stakeholders recommended clinical training before mentorship because mentees’ pre-service clinical knowledge and fundamental skills were insufficient.

##### Inadequate support for mentors and insufficient dose

A common area for improvement suggested by key stakeholders was the need for greater mentor support. Mentoring was perceived as a service provided by senior physicians or as an extracurricular activity. However, mentoring requires training, adequate compensation, recognition, and backup support at their home hospitals. The lack of support resulted in some attrition of mentors, affecting continuity. Mentees desired more time; mentors explained that logistics such as transportation arrangements made it difficult for them to stay longer.

#### Organizational context

##### Challenging implementation context

The greatest barrier to implementation discussed was the challenging implementation context including workforce, infrastructure, and organizational culture. One mentee explained that while the goal was to increase surgical volume, all surgeries would be cancelled if their only anesthetist was ill. Mentors described challenges with high turnover; nurses rotated through ORs and were transferred constantly. The lack of supplies and equipment made it difficult for mentees to implement best practices. Finally, mentors and external stakeholders explained that some facilities had weak safety cultures; they did not discuss cases as a team, review data, or use evidence-based safety practices consistently.

##### Lack of a clear understanding of the mentorship intervention

A few interviewees explained a lack of understanding about the mentorship intervention. Some mentees thought the role of mentors was to mobilize resources; others feared consequences of mistakes because they did not understand the purpose of mentorship, and a few mentees expected incentives for additional work. Mentors experienced compassion fatigue as expectations for resources were high and unrealistic.

### Perceived impact

All mentees (100%, *n* = 24/24) experienced the intervention as positive, 79% (*n* = 19/24) were satisfied and 87% (*n* = 21/24) supported continuing the intervention (Additional File 4). The majority of mentees said their surgical practices changed due to mentorship (84%, *n* = 21/25) and that their hospital made changes as a result of the program (76%, *n* = 19/25) (Additional File 5). Over 70% of mentees reported that confidence (75%, *n* = 18/24), SSC implementation (75%, *n* = 18/24), and improved job satisfaction (71%, *n* = 17/24) all improved “to a great extent”. (Fig. 2). The qualitative results provided deeper insights about the perceived impact of mentorship.

#### Safer and more frequent provision of surgical care

Mentees consistently discussed how mentorship helped implement evidence-based safety practices. Before the intervention, these practices were not used or performed consistently causing poor surgical care and outcomes. Participants believed standardization of these practices, better teamwork and communication, and improvements in the surgical ecosystem helped mentees improve surgical volume, outcomes, safety culture, and communication with patients. Mentees attributed this to mentorship because mentors continuously emphasized, demonstrated, and encouraged best practices.

#### Establishment of collegial bonds between mentees and mentors

A few mentees and mentors noted collegial relationships lasted beyond onsite interactions. Mentors continued to consult difficult cases after mentorship and encouraged mentees to seek advice when needed. Mentors said this helped reduce referrals out and provided opportunity to continue building confidence in mentees.

#### Empowered mentees

Mentees commonly conveyed a greater sense of empowerment. They explained that, previously, challenges in providing surgical care at facilities were not their responsibility, but now felt ownership of problems and wanted to solve them. Mentors discussed mentees had greater confidence and agency. This sense of empowerment went beyond individuals to teams taking ownership for the care of patients. Mentees perceived leadership was more invested in improving surgical services and outcomes of surgical care.

#### Inculcation of a culture of continuous learning

Participants highlighted that mentorship inculcated a culture of continuous learning. Mentors demonstrated the data collection systems, explained their importance, trained mentees in their use, and ensured they were followed. Mentees reported this helped identify mistakes and strategize on areas of improvement. Mentors reported mentoring allowed them to learn; one discussed the importance of mentees and mentors learning from each other by putting the patient at the center. Another explained mentorship allowed them to better understand the context of rural facilities. This impact was significant and inspired others at the national level.

## Discussion

### Statement of principal findings

Our results suggest mentorship is a promising strategy for building capacity of surgical providers in resource-constrained settings. Participants perceived safer care, increased surgical volume, amicable bonds between mentees and mentors, empowered mentees, and a culture of continuous learning as impacts of the intervention. Over two-thirds of mentees reported an increase in confidence and job satisfaction. The intervention’s holistic approach, effective mentee-mentor relationships, and receptive climate contributed to its success.

### Interpretation of results within the context of the wider literature

In LMICs, research on the role of mentorship in surgery is at a nascent stage [53, 54] and surgical mentorship program implementation has not been sufficiently analyzed to guide policy and practice; this is important to successfully design and implement mentorship programs [6]. We found results from implementing SS2020 mentorship in Ethiopia similar to Tanzania [5], with strong support from mentees and similar perceived impacts, including safer care, strengthened surgical ecosystems, increased confidence, and stronger learning cultures in both countries. Common supportive features included a holistic approach, multidisciplinary mentorship team, psychologically safe relationships, mentors that were friendly, accessible, and understanding of the local context, and a receptive implementation climate. Common challenges included resource constraints, inadequate mentor support, insufficient dose, and resistance to change.

### Implications for policy, practice and research

We offer lessons to intervention designers, policy makers, and practitioners about optimizing surgical mentorship in LMICs (Table 3).

**Table 3:** Lessons learned for future implementation of surgical mentorship programs in LMICs

Key Implementation Features	Lessons
**Key intervention characteristics**	To improve access to safe, high-quality surgery in low resource settings, mentorship should take a **holistic approach** by building technical and non-technical skills in surgical providers and strengthening the surgical ecosystem.
	A **multidisciplinary team approach** provides a comprehensive approach to mentorship (e.g. an anesthetist mentoring an anesthetist), and also facilitates teamwork and collective learning among mentees.
	An environment of **psychological safety** where mentees can ask questions, raise concerns or admit mistakes without fear of consequences is important for learning. Mentees value relationships with mentors that are friendly, non-hierarchical, nonjudgmental and safe.
	**Clinical training** prior to mentorship ensures standardization of evidence-based practices and provides a base of knowledge for successful implementation. **Side-by-side surgical coaching** at the mentee’s facility is a practical and efficient way of learning for mentees within their own context.
	**Innovations to increase dose** such embedded mentorship, coupled facilities, and focal mentors within facilities should be explored. Blended models which combine in-person and virtual options such as tele-mentoring, WhatsApp and SMS can reinforce learning, strengthen relationships, and provide real-time support
	The **goals, scope, and performance targets** of the mentorship intervention should be defined in conjunction with mentees, mentors, facility leaders, and regional authorities to facilitate buy-in and a common understanding.
	Mentorship must be a **managed process** for success.
	The design and implementation of the intervention should be guided by **regular feedback** from mentees and mentors. Feedback can be used to inform training and skills strengthening for mentors, to refine implementation, or to adapt the intervention, and can be obtained through mechanisms such as debriefing at the end of the mentorship visit, joint learning sessions, or surveys.
	Data is necessary in understanding achievements and challenges. A **monitoring and evaluation plan** with key performance indicators related to inputs, provider behaviors, and patient outcomes should be identified, targets should be set, and reports should be shared.
	A **model of surgical mentorship** can be developed at a small number of sites and then scaled up.
**Supportive organizational Context**	**A situation analysis** helps to understand inner context, priorities for strengthening surgical services, leadership support, and readiness for change. It can help to identify priorities and barriers to implementation and tailor the intervention for success.
	**Leaders who are engaged** in the mentorship intervention can communicate objectives, align incentives, remove barriers, address resistance, and promote collective learning through data use, simulations, or debriefings.
	**Regional authorities should participate in trainings and experience sharing forums** to understand the challenges faced by surgical teams and facilitate solutions.
	Improving access to safe, high-quality surgical care requires adequate resources. **Resources to must be available** to ensure the success of the mentorship intervention.
	**Mentors require resources** for capacity building, compensation, back up to allow time away from regular responsibilities, logistical support, guidelines, and recognition.
	Sustainability of the mentorship program requires **policy support, alignment with national strategy, integration within the health system, and development of a culture of mentorship.**
	Mentorship requires the **collaboration, engagement, and support from key stakeholders** such as government, professional societies, academic medical institutions, and senior surgical professionals. These key stakeholders can provide essential technical and professional support to the mentorship process, promote mentorship as an approach, and mobilize professionals and resources.

#### Intervention characteristics

The intervention’s holistic approach was critical. The context was challenging, with limited workforce, resource constraints, and a weak safety culture. A single vertical intervention would not have succeeded; instead, the entire surgical system required intervention through building technical and non-technical skills, strengthening infrastructure, and changing the culture [55–57].

Mentees valued psychological safety with mentors where they could ask questions, speak up, or admit mistakes. Studies have linked psychological safety with improved learning and performance [58–61]; future interventions should focus on fostering this quality in mentor training for optimal effectiveness.

Mentees prioritized clinical knowledge and skills the most, similar to findings in Tanzania, but the discrepancy between what was desired versus received differed. In Ethiopia, 92% of mentees wanted clinical skills but only 44% said it was received compared to Tanzania (96% and 76%, respectively). Fewer Ethiopian mentees (39%) reported their clinical skills improved to a great extent compared to Tanzanian mentees (74%). The combined effect of training and mentorship may be more effective [62] and future surgical mentorship programs should consider clinical training before mentorship and clinical mentorship during visits [63, 64].

Innovative and cost-effective options for increasing dose should be explored. Research suggests in-person mentorship is valuable for building relationships, understanding local context, and learning tacit knowledge [55]. Approaches include embedded mentorship, where mentors work within a mentee facility for a defined period [65, 66]; a focal mentor, where an experienced provider within the facility provides mentorship [67]; and coupled facilities, where a higher-performing facility mentors a lower-performing facility [32, 53]. Blended mentorship, which combines in-person and virtual options such tele-mentoring, WhatsApp, and SMS can increase dose, complement learning, strengthen relationships, and provide real-time support [68–73].

Learning and adaptation of the mentorship intervention is important for improving the intervention’s design. Feedback from mentees and mentors can inform adaptation; a monitoring plan with output indicators regarding mentoring visits, provider behaviors, and patient outcomes can provide insight about impact [74]. Through learning and adaptation, a model of surgical mentorship can be successfully scaled up.

#### Organizational context

A receptive implementation climate was key for the intervention’s success. A situation analysis helped tailor the intervention by understanding context, identifying priorities, gaining leadership support, and preparing for change [75, 76]. However, adequate resources are needed for implementation. While mentors addressed resource constraints, only 20% of mentees perceived those resources were mobilized, suggesting resources were a significant challenge. Providing resources has potential to improve adherence to safety standards [77, 78] and access [79–81].

Institutionalizing mentorship requires policy support, alignment with national reforms, integration within health systems, and, over time, a culture of mentorship. Mentorship requires collaboration, engagement, and support from stakeholders such as government, professional societies, academic medical institutions, and senior surgical professionals; they can provide technical and professional support, promote mentorship, and mobilize resources [38]. Regional authorities should be involved from inception to understand challenges faced by surgical teams.

#### Future research

To understand the impact of mentorship on surgical access and quality and its sustainability, experimental and longitudinal research design should be considered in the future. Further research is necessary to understand factors such as optimal dose, the importance of readiness for change for success [82], and which contextual factors, resources, and structures are important. Research can inform the optimal design of the intervention, how to adapt it based on the implementation context, uncover best practices, and measure impact [83–86]. This research can support effective policy and practice by elucidating how and where resources should be spent, and can be used as the basis for effectively scaling up for impact.

### Strengths and limitations

The key strength of our study is its mixed-methods design and primary data collection from participants, but there were limitations. We could not collect data from SS2020 sites in the Amhara region due to conflict; our findings need to be confirmed with a larger sample in more diverse contexts and follow-up. Our survey should be refined for future research based on the research question and conceptual framework. Respondents may have given more positive responses, however, using an experienced qualitative researcher and collecting data from different respondents at each site may have minimized bias [87]. Our findings may have been confounded by participants’ experiences with other SS2020 interventions.

## Conclusion

We conducted a mixed-methods evaluation regarding the experiences of mentees, mentors, hospital leaders, and key stakeholders to generate lessons on optimizing the design and implementation of surgical mentorship interventions in resource-constrained settings. Our findings suggest mentorship may build capacity in surgical providers, increase confidence and job satisfaction, and instill a culture of continuous learning in low-resource settings. A holistic approach, a receptive implementation climate, effective mentee-mentor relationships, adaptation through user-feedback, and policy support to institutionalize and build a culture of mentorship are important to the intervention’s success.

## Supplementary Information

**Additional file 1.** Ethiopia mentorship survey.

**Additional file 2.** Interview protocols.

**Additional file 3.** Mentor and mentoring relationships and characteristics.

**Additional file 4.** Overall experience, satisfaction and recommendations for continuation.

**Additional file 5.** Percentage of respondents reporting changes as a result of mentorship.

## Data Availability

The dataset analyzed during the current study is available from the corresponding author on reasonable request.

## Abbreviations

FMOH: Federal Ministry of Health
IESO: Integrated Emergency Surgical Officers
OR: Operating Room
RHB: Regional Health Bureau
SaLTS: Saving Lives through Safe Surgery
SS2020: Safe Surgery 2020
SSC: Surgical Safety Checklist
