# Supplementary material for: Implementing surgical mentorship in a resource-constrained context: a mixed methods assessment of the experiences of mentees, mentors, and leaders, and lessons learned
Source: BMC Med Educ. 2022 Aug 31;22:653. doi: 10.1186/s12909-022-03691-2 (PMC9434847; doi:10.1186/s12909-022-03691-2)
Supplement: Supplementary file 2 — Additional file 2. Interview protocols. [file 12909_2022_3691_MOESM2_ESM.docx]

**Additional File 2 - Interview Protocols**

# Safe Surgery 2020 - Ethiopia Mentorship Program – Surgical Team Members Interview

The goal of the Safe Surgery 2020 initiative is to reduce preventable deaths from surgically- treatable conditions by improving safe, timely, and affordable access to surgical care. We are seed funded by the GE Foundation, hosted by Dalberg, and implemented by Jhpiego, Assist International, Harvard Medical School’s Program in Global Surgery and Social Change, and the G4 Alliance.

We are conducting interviews of participants involved in the Safe Surgery 2020 Initiative’s mentoring intervention to obtain feedback on the mentorship program. Your participation will help us to understand your perception of the mentorship program related to areas of mentoring, your experience with your mentor, the impact made by the mentorship program, and how the program can improve.

Completing this interview should take about an hour. We will take notes and record our conversation so that we can review it. We will not share anything you say in an identifiable fashion. No one at this facility will have access to your responses; they will only see summary reports. Interview content will be analyzed by researchers in the Program in Global Surgery and Social Change at Harvard Medical School.

Participation in this interview is completely voluntary. If you choose not to participate it will have no effect on your employment and no one will know that you declined to participate. Participants will not receive any compensation for participating in this interview. If you do not understand a question, please let me know and I will explain it. You may choose to not answer any questions that you do not feel comfortable answering.

**Background**

1. Can you tell me about your position at the hospital? How many years have you worked in this position?
2. Did you participate in Jhpiego’s leadership and mentorship training? (Have you had a visit from a mentor in the last 6 months? How many visits have you had?)

# Safe Surgery 2020 Initiative Mentoring Intervention

1. Can you describe your understanding of what mentorship is? How is it different than supervision?
2. In your own words, can you describe the mentorship intervention? (Prompt for process, timeline, structure, SS2020 purpose)
3. In what ways do you think the mentoring program is contributing to the strengthening surgical and anesthetic services? (e.g. Facility benefits, provider skill benefits)
4. Can you describe the goals and priorities of the program? Do they align with you own?

# Areas of mentoring support

1. In what areas have you received mentoring support?
   1. Can you provide an example or description of each area you have received support in?
   2. Of the areas of mentoring discussed, which would you say are the most and least valuable and why?
2. In what areas are you aware of mentoring support outside the facility?
   1. Can you provide an example or description for each area you have received support in?
   2. Of the areas of mentoring discussed, which would you say are the most and least valuable and why?

# Perceptions of program, mentors, mentor relationships, and experiences

1. Can you describe your overall experience with the mentorship program?
2. Can you describe some characteristics that made your mentor effective? (Prompt for traits and awareness of local context and conditions)
3. Can you describe your relationship with your mentor? (Prompt for relationship dynamics, style of mentoring, comfort and trust). What worked best for you and your mentor? (e.g. style of mentorship (if not mentioned before, means of communication, feedback)
4. Can you describe the general attitude around adopting the mentoring program in your facility? (Prompt for acceptance or resistance, culture change, culture of mentoring)

# Difference made by mentoring

1. What has been the overall impact of the program? (Prompt for impact related to clinical mentoring, enabling environment, advocacy and resource mobilization, leadership and management)
2. What changes have been made as a result of the mentoring program? (e.g. changes in your professional practice, changes at the facility level)
3. Has the program had any impact on your personal life? (e.g. stress reduction, work-life balance, improved workplace relationships)
4. What changes would you like to see that have not been affected by the program? (e.g. personally, professionally, work environment-related)

# Strengths, challenges, areas for improvement, and lessons learned

1. What would you say are the strengths of the program? (e.g. What did you like best? Communication style (in-person, text, call), mentorship style (side by side, one on one))
2. What would you say are the challenges associated with the program? (e.g. personal challenges faced during the program, facility challenges, foreseen challenges with sustainability or continuation of the program
3. What made the program work well and what did not make the program work well? (probe for facilitators and barriers)
   1. Are these due to the program?
   2. How can the barriers be addressed?
4. What are the areas in which the program could improve?
5. Can you describe some of the lessons learned from the program? (e.g. personally, professionally, at the facility level)

# Satisfaction

1. Are you satisfied with the program? Why or why not?
2. In what ways are you or are you not satisfied with yourself after participating in this program? (e.g. confidence, self-sufficiency, job satisfaction)
3. Do you support the continuation of the mentorship program? Why or why not?

# Closing remarks

1. Do you have any further questions, concerns, or comments for our team regarding the mentorship program?

# Safe Surgery 2020 - Ethiopia Mentorship Program – Mentor Interview

The goal of the Safe Surgery 2020 initiative is to reduce preventable deaths from surgically-treatable conditions by improving safe, timely, and affordable access to surgical care. We are seed funded by the GE Foundation, hosted by Dalberg, and implemented by Jhpiego, Assist International, Harvard Medical School’s Program in Global Surgery and Social Change, and the G4 Alliance.

We are conducting interviews of participants involved in the Safe Surgery 2020 Initiative’s mentoring intervention to obtain feedback on the mentorship program. Your participation will help us to understand your perception of the mentorship program related to areas of mentoring, your experience with your mentor, the impact made by the mentorship program, and how the program can improve.

Completing this interview should take about an hour. We will take notes and record our conversation so that we can review it. We will not share anything you say in an identifiable fashion. No one at this facility will have access to your responses; they will only see summary reports. Interview content will be analyzed by researchers in the Program in Global Surgery and Social Change at Harvard Medical School.

Participation in this interview is completely voluntary. If you choose not to participate it will have no effect on your employment and no one will know that you declined to participate. Participants will not receive any compensation for participating in this interview. If you do not understand a question, please let me know and I will explain it. You may choose to not answer any questions that you do not feel comfortable answering.

**Background**

1. Can you tell me about your position at the hospital? How many years have you worked in this position?
2. Did your facility participate in Jhpiego’s leadership and mentorship training? If so, how many mentoring visits would you say you have done?

# Safe Surgery 2020 Initiative Mentoring Intervention

1. Can you describe your understanding of what mentorship is? How is it different than supervision?
2. In your own words, can you describe the mentorship intervention? (Prompt for process, timeline, structure, SS2020 purpose)
   1. Can you speak to the timeline and structure of the program? What about the duration and intensity of visits and ratio of mentors to surgical staff?
   2. Have your mentor visits been consistent over the last six months?
3. Can you describe the goals and priorities of the program? Do they align with your own as a mentor?
4. In what ways do you think the mentoring program is contributing to strengthening of surgical and anesthetic services in your facility? (e.g. Facility benefits, provider skill benefits)

# Areas of mentoring support

1. Can you describe the areas of mentoring that the program covers in the facility? What about outside of the facility?
   1. In which areas have you provided the most mentoring support? The least support?
   2. In which areas would you say surgical staff need the most support?
   3. Which areas would you say are the most valuable to your facility? Which areas would you say are the least valuable?

# Perceptions of program, mentors, mentor relationships, and experiences

1. Can you describe your overall experience with the mentorship program?
2. Why did you want to be a mentor?
3. How would you describe yourself as a mentor? What characteristics do you think your surgical staff would use to describe you?
4. How would you describe your relationships with the surgical staff you mentor?
   1. How comfortable were you in providing mentoring support?
   2. Did you feel confident in your skills?
   3. Were surgical staff members willing to seek help from you and admit mistakes?
5. What was your style of mentoring?
   1. Can you describe the ways in which you communicate with the surgical staff?
   2. How do you provide feedback to one another?
6. Can you describe the general attitude around adopting the mentoring program in your facility?
   1. How did the mentors react to their new responsibility? Did you feel the same way?
   2. How did the surgical staff react to having a mentor?

# Difference made by mentoring

1. How would you describe the overall impact of the program? (Prompt for impact related to clinical mentoring, enabling environment, advocacy and resource mobilization, leadership and management)
2. What changes have been made as a result of the mentoring program? Have you seen changes in facility staff or system processes?
3. What has been the most significant change or impact you have seen in your surgical staff as a result of this program?
4. Has the program had any impact on your personal life? (Prompt for stress reduction, better relationships in the workplace, a sense of purpose)
5. Did the mentorship program meet your expectations? Why or why not? Would you consider the program successful?
6. What changes would you like to see that have not been affected by the program? (e.g. management, work environment-related, improved quality of care)

# Strengths, challenges, areas for improvement, and lessons learned

1. From your perspective as a mentor, what would you say are the strengths of the program?
2. What would you say are the challenges associated with the program? (e.g. facility challenges, foreseen challenges with sustainability or continuation of the program)
3. What made the program work well and what did not make the program work well? (probe for facilitators and barriers)
   1. Are these due to things related to the program?
   2. How can the barriers be addressed?
4. What are the areas in which the program could improve?
5. Can you describe some of the lessons learned from the program? (e.g. personally, professionally, at the facility level)

# Satisfaction

1. Are you satisfied with the program? Why or why not?
2. If satisfied with the program, can you provide a few examples of what you are satisfied with? (Prompt for confidence in clinicians, reassurance)
3. Do you support the continuation of the mentorship program? Why or why not?

# Closing remarks

1. Do you have any further questions, concerns, or comments for our team regarding the mentorship program?

# Safe Surgery 2020 - Ethiopia Mentorship Program – Hospital Leaders Interview

The goal of the Safe Surgery 2020 initiative is to reduce preventable deaths from surgically- treatable conditions by improving safe, timely, and affordable access to surgical care. We are seed funded by the GE Foundation, hosted by Dalberg, and implemented by Jhpiego, Assist International, Harvard Medical School’s Program in Global Surgery and Social Change, and the G4 Alliance.

We are conducting interviews of participants involved in the Safe Surgery 2020 Initiative’s mentoring intervention to obtain feedback on the mentorship program. Your participation will help us to understand your perception of the mentorship program related to areas of mentoring, your experience with your mentor, the impact made by the mentorship program, and how the program can improve.

Completing this interview should take about an hour. We will take notes and record our conversation so that we can review it. We will not share anything you say in an identifiable fashion. No one at this facility will have access to your responses; they will only see summary reports. Interview content will be analyzed by researchers in the Program in Global Surgery and Social Change at Harvard Medical School.

Participation in this interview is completely voluntary. If you choose not to participate it will have no effect on your employment and no one will know that you declined to participate. Participants will not receive any compensation for participating in this interview. If you do not understand a question, please let me know and I will explain it. You may choose to not answer any questions that you do not feel comfortable answering.

**Background**

1. Can you tell me about your position at the hospital? How many years have you worked in this position?
2. Did your facility participate in Jhpiego’s leadership and mentorship training?

# Safe Surgery 2020 Initiative Mentoring Intervention

1. Can you describe your understanding of what mentorship is? How is it different than supervision?
2. In your own words, can you describe the mentorship intervention? (Prompt for SS2020 purpose process, timeline, structure)
3. Can you describe the goals and priorities of the program?
4. In what ways do you think the mentoring program is contributing to the strengthening surgical and anesthetic services in your facility? (e.g. Facility benefits, provider skill benefits)

# Areas of mentoring support

1. Can you describe the areas of mentoring that the mentors provide to you and your hospital’s surgical team?
   1. In which areas would you say mentors are providing the most support? The least support?
   2. In which areas would you say are the most valuable to your facility? Which areas would you say are the least valuable?

# Perceptions of program, mentors, mentor relationships, and experiences

1. Can you describe your overall experience with the mentorship program?
2. Can you describe some characteristics that made the mentors in your facility effective?
3. How would you describe the relationships between the mentors and surgical staff?
   1. How comfortable did the mentors seem in their role?
   2. Were mentors confident in their skills?
   3. Were surgical staff members willing to seek help and admit mistakes to mentors?
4. In which ways are you involved in the mentoring process? How do you support it?
5. Can you describe the general attitude around adopting the mentoring program in your facility? (Prompt for acceptance or resistance, culture change, culture of mentoring)
6. Has the mentorship program met your expectations? Why or why not? Would you consider the program successful?

# Difference made by mentoring

1. What has been the overall impact of the program? (Prompt for impact related to clinical mentoring, enabling environment, advocacy and resource mobilization, leadership and management)
2. What changes have been made as a result of the mentoring program?
   1. What changes have been made at a hospital level (including system processes)
   2. Have you seen changes in the surgical team or processes related to surgery?
3. Did the mentoring program meet your expectations? Why or why not?
4. What changes would you like to see that have not been affected by the program? (e.g. management, work environment-related, improved quality of care)

# Strengths, challenges, areas for improvement, and lessons learned

1. From your perspective, what would you say are the strengths of the program?
2. What would you say are the challenges associated with the program? (e.g. facility challenges, foreseen challenges with sustainability or continuation of the program)
3. What made the program work well and what did not make the program work well? (probe for facilitators and barriers)
   1. Are these due to things related to the program?
   2. How can the barriers be addressed?
4. What are the areas in which the program could improve?
5. Can you describe some of the lessons learned from the program?

# Satisfaction

1. Are you satisfied with the program? Why or why not?
2. If satisfied with the program, can you provide a few examples of what you are satisfied with? (Prompt for confidence in clinicians, reassurance)
3. Do you support the continuation of the mentorship program? Why or why not?

# Closing remarks

1. Do you have any further questions, concerns, or comments for our team regarding the mentorship program?

# Safe Surgery 2020 - Ethiopia Mentorship Program – Key stakeholder interview

The goal of the Safe Surgery 2020 initiative is to reduce preventable deaths from surgically-treatable conditions by improving safe, timely, and affordable access to surgical care. We are seed funded by the GE Foundation, hosted by Dalberg, and implemented by Jhpiego, Assist International, Harvard Medical School’s Program in Global Surgery and Social Change, and the G4 Alliance.

We are conducting interviews of participants involved in the Safe Surgery 2020 Initiative’s mentoring intervention to obtain feedback on the mentorship program. Your participation will help us to understand your perception of the mentorship program related to areas of mentoring, your experience with your mentor, the impact made by the mentorship program, and how the program can improve.

Completing this interview should take about an hour. We will take notes and record our conversation so that we can review it. We will not share anything you say in an identifiable fashion. No one at this facility will have access to your responses; they will only see summary reports. Interview content will be analyzed by researchers in the Program in Global Surgery and Social Change at Harvard Medical School.

Participation in this interview is completely voluntary. If you choose not to participate it will have no effect on your employment and no one will know that you declined to participate. Participants will not receive any compensation for participating in this interview. If you do not understand a question, please let me know and I will explain it. You may choose to not answer any questions that you do not feel comfortable answering.

**Background**

1. Can you tell me about your role with the mentoring program in Ethiopia?
2. How long did you work with the mentoring program or initiative?

# Safe Surgery 2020 Initiative Mentoring Intervention

1. Can you tell me about the history of the mentoring intervention? Why did Ethiopia need this program to strengthen surgical services?
2. How did you conceptualize the mentoring intervention?
3. How did you go about planning the mentoring intervention?

# Mentoring process

1. What are the goals of the mentoring intervention?
2. Can you describe the mentoring process?
3. What are the resources required to establish a mentoring program?
4. What areas of mentoring support are provided to the surgical team and facility leaders?
5. Clinical mentoring
6. Resource mobilization
7. Community engagement
8. Can you describe the key elements of a well-functioning, sustainable mentoring system?

# Perceptions of the program and scale-up

1. How do you see mentoring is making a difference?
2. What are some of the lessons learned from this program?
3. What are the areas for improvement within the program?
4. What would you say are the challenges with implementing the program in other facilities in Ethiopia?
5. Do you support the continuation and scale-up of the mentorship program?
6. Why or why not?
7. What would you do differently if the program were to be implemented in another region?
8. What does your vision for the mentorship program look like?

# Closing remarks

1. Do you have any further questions, concerns, or comments for our team regarding the mentorship program?
