# Supplementary material for: Implementing surgical mentorship in a resource-constrained context: a mixed methods assessment of the experiences of mentees, mentors, and leaders, and lessons learned
Source: BMC Med Educ. 2022 Aug 31;22:653. doi: 10.1186/s12909-022-03691-2 (PMC9434847; doi:10.1186/s12909-022-03691-2)
Supplement: Supplementary file 3 — Additional file 3. Mentor and mentoring relationships and characteristics. [file 12909_2022_3691_MOESM3_ESM.docx]

**Additional File 3 – Mentor and Mentoring Relationships and Characteristics**
