# Supplementary figures and images for: Implementing surgical mentorship in a resource-constrained context: a mixed methods assessment of the experiences of mentees, mentors, and leaders, and lessons learned
Source: BMC Med Educ. 2022 Aug 31;22:653. doi: 10.1186/s12909-022-03691-2 (PMC9434847; doi:10.1186/s12909-022-03691-2)

**Additional File 4 – Overall Experience, Overall Satisfaction, and Support for Continuation**


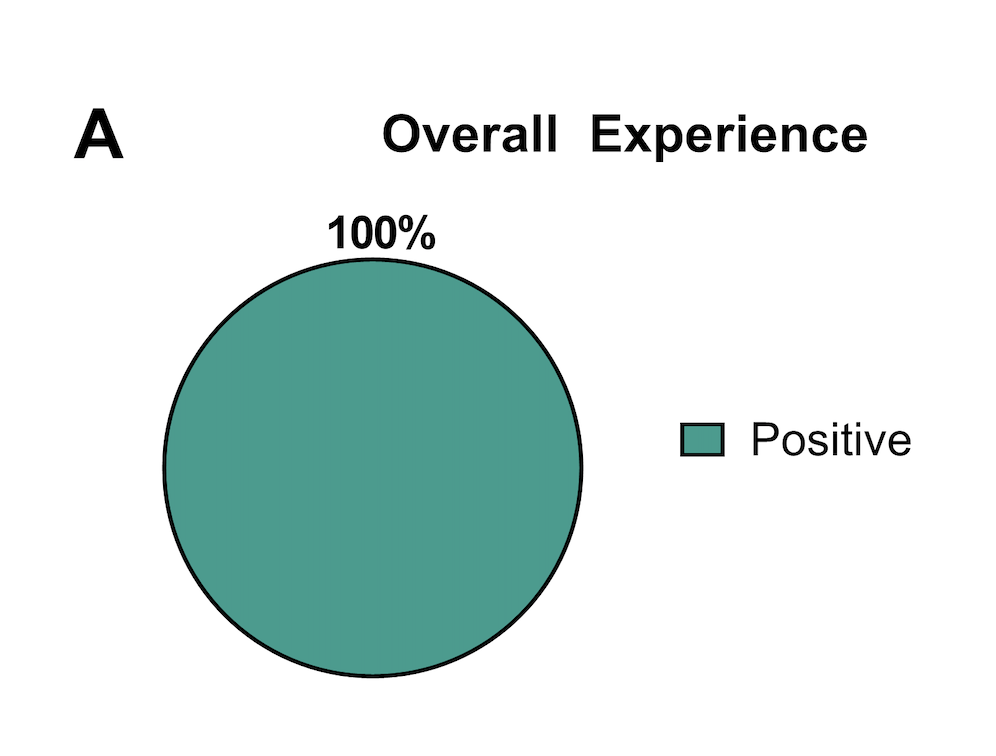


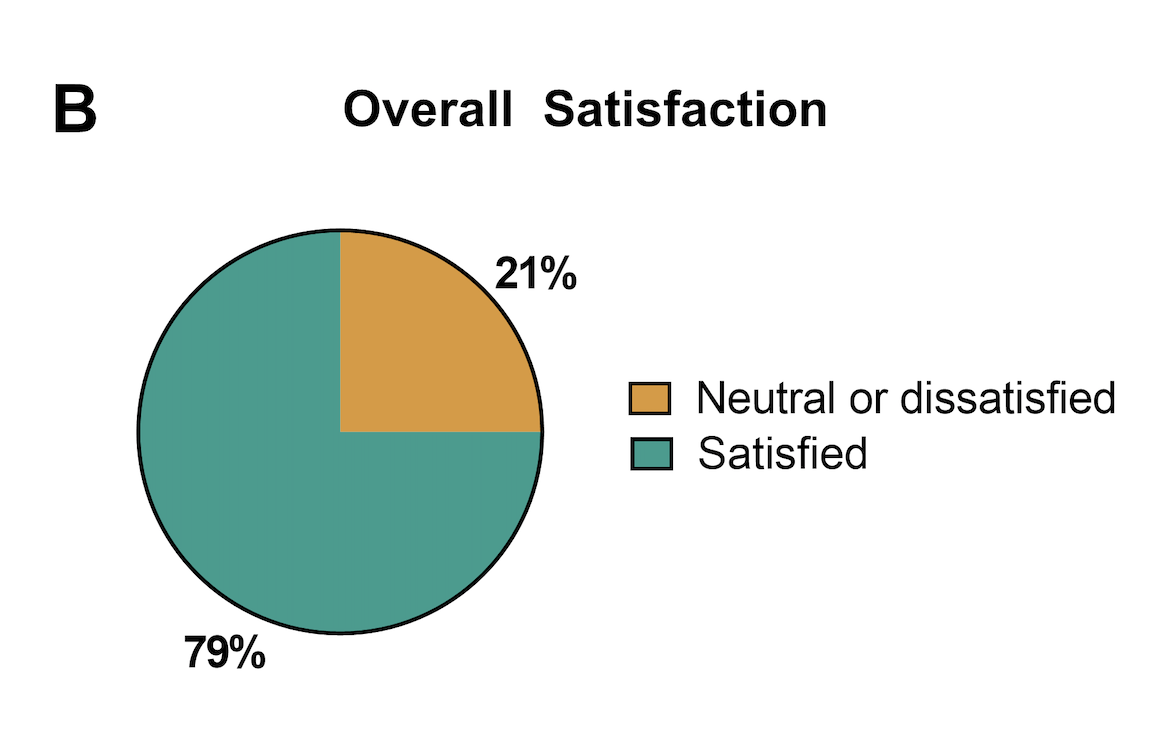


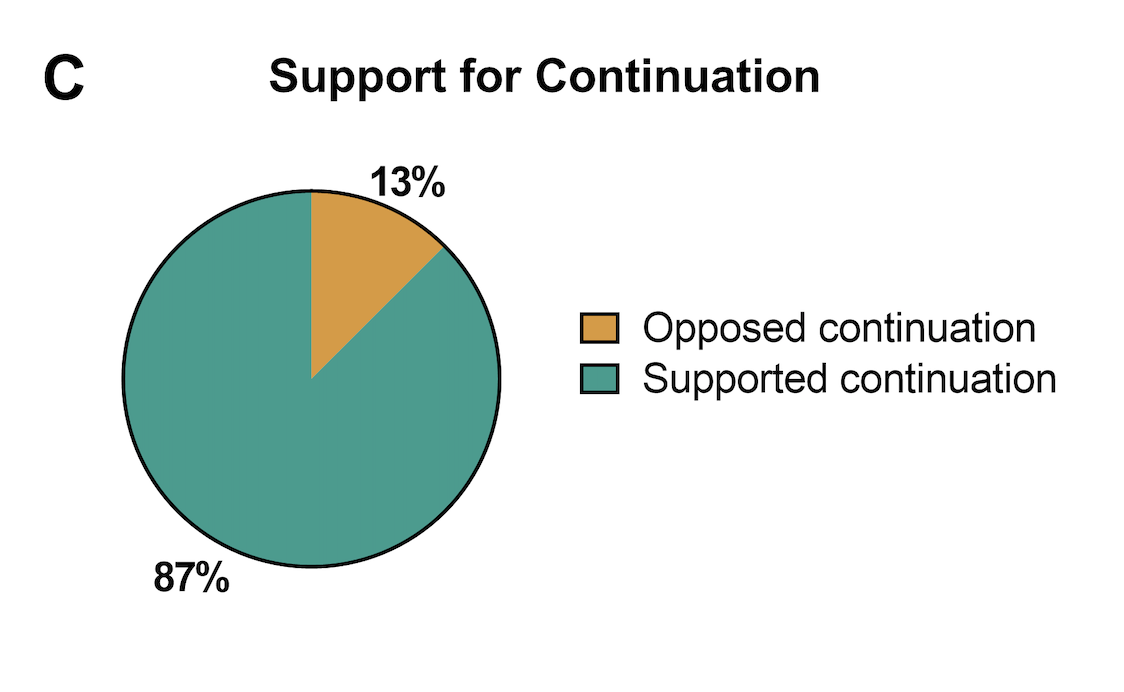

Supplement: Supplementary file 4 — Additional file 4. Overall experience, satisfaction and recommendations for continuation. [file 12909_2022_3691_MOESM4_ESM.docx]

**Additional File 5 – Percentage of Respondents Reporting Changes As a Result of Mentorship**


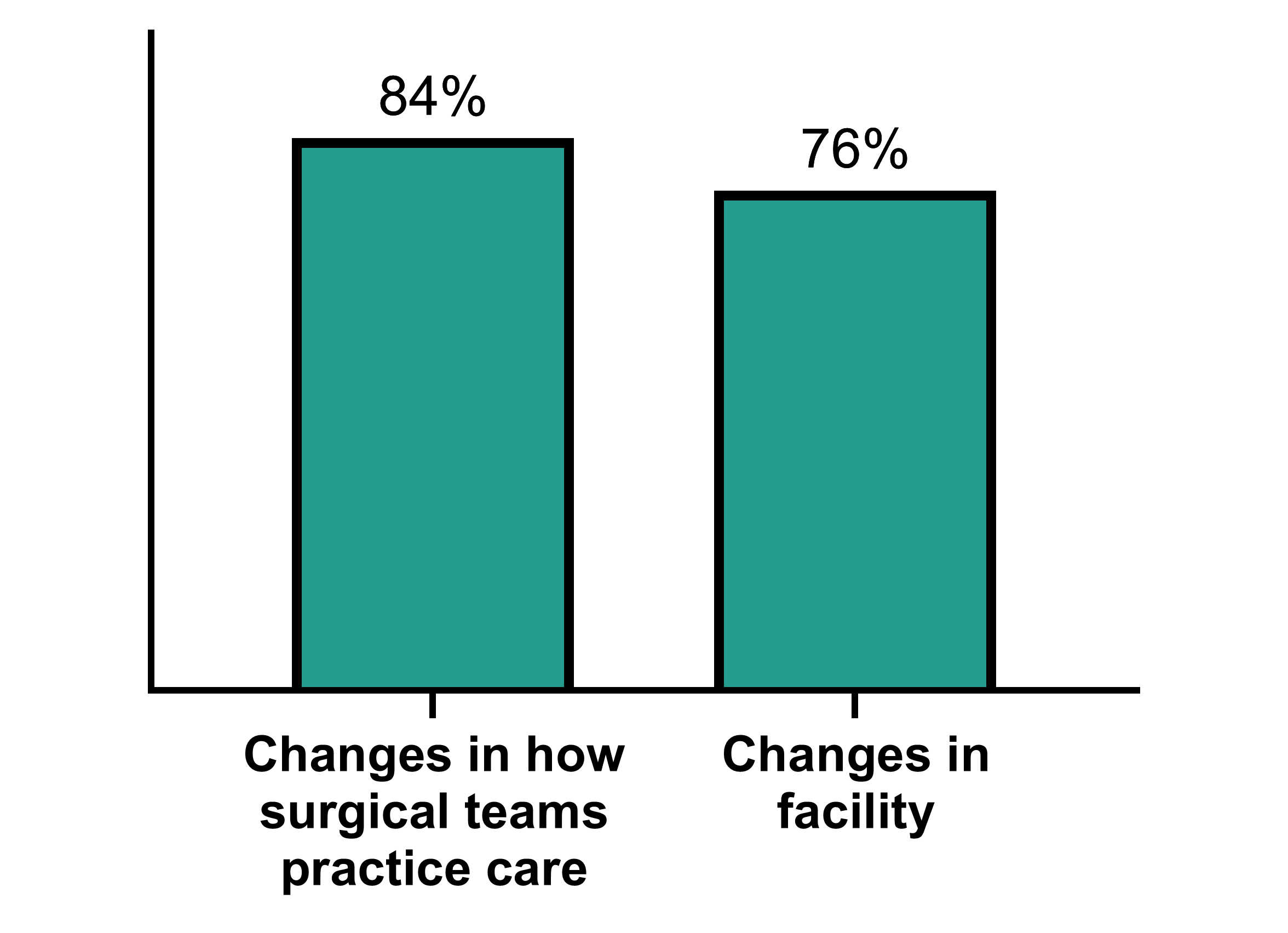

Supplement: Supplementary file 5 — Additional file 5. Percentage of respondents reporting changes as a result of mentorship. [file 12909_2022_3691_MOESM5_ESM.docx]
